# Comprehensive plastid phylogenomics reveal the phylogeny of *Amomum* s.l. (Zingiberaceae) from Peninsular Malaysia

**DOI:** 10.3897/phytokeys.276.192768

**Published:** 2026-06-12

**Authors:** Fathmath Shaman Fareed, Nallammai Singaram, Ahmad Zuhairi Abdul Malek, Amalia Mohd Hashim, Yen Yen Sam, Ooi Im Hin

**Affiliations:** 1 School of Biosciences, Faculty of Health and Medical Sciences, Taylor’s University, 47500, Subang Jaya, Selangor, Malaysia School of Biosciences, Faculty of Health and Medical Sciences, Taylor’s University Selangor Malaysia; 2 Centre for Active Living, Taylor’s University, 47500 Subang Jaya, Selangor, Malaysia Centre for Active Living, Taylor’s University Selangor Malaysia; 3 Halal Product Research Institute, University Putra Malaysia, 43400 Serdang, Selangor, Malaysia Halal Product Research Institute, University Putra Malaysia Selangor Malaysia; 4 Department of Microbiology, Faculty of Biotechnology and Biomolecule Sciences, Universiti Putra Malaysia, 43400, Serdang, Selangor, Malaysia Department of Microbiology, Faculty of Biotechnology and Biomolecule Sciences, Universiti Putra Malaysia Selangor Malaysia; 5 Forest Research Institute Malaysia, 52109, Kepong, Selangor, Malaysia Forest Research Institute Malaysia Selangor Malaysia; 6 The Habitat Penang Hill, C7G9+69, Bukit Bendera, 11300 Bukit Bendera, Penang, Malaysia The Habitat Penang Hill Penang Malaysia

**Keywords:** *Amomum* s.l., chloroplast genes, phylogenomics, plastid, species delimitation, transcriptome

## Abstract

The Zingiberaceae family represents one of the most diverse monocot lineages in tropical Asia; however, the species delimitation of *Amomum* s.l. remains challenging due to morphological convergence and limited molecular evidence. This study analyzed transcriptomic data from seven *Amomum* s.l. species in Peninsular Malaysia to evaluate their evolutionary relationships and examine variation across chloroplast genes. RNA sequencing and *de novo* assembly identified 85 chloroplast protein-coding genes, of which 43 complete genes were retained for subsequent analyses. Comparative assessment revealed conserved genome features, including GC content ranging between 37.4% and 38.0%, alongside lineage-specific divergence in codon usage bias and expression profiles. Heatmap clustering of normalized gene expression patterns indicated distinct transcriptional signatures, particularly in *A. elan*, suggesting species-specific regulatory divergence. Nucleotide diversity (*π*) analysis identified hotspots in *ndhJ*, *psbT*, *psbZ*, and *rps14*, highlighting their potential as molecular markers for improving species delimitation, whereas core genes such as *rbcL* and *psbA* remained highly conserved, reducing their effectiveness in distinguishing species-level variation. Phylogenetic reconstruction using Bayesian inference (BI) and maximum likelihood (ML) approaches, based on 43 concatenated chloroplast genes (28,794 bp), supported the paraphyly of *Amomum* s.l., with Peninsular Malaysian species distributed across multiple clades. *Amomum trilobum* showed a closer association with *Wurfbainia* than with *Amomum*–*Alpinia*, suggesting the possible transfer of the species to *Wurfbainia* with further taxonomic evaluation. These findings highlight the important roles of genomic and morphological datasets in understanding the evolutionary relationships in *Amomum* s.l. and offer new molecular insights into the evolution of Zingiberaceae in Peninsular Malaysia.

## Introduction

The Zingiberaceae family, which belongs to the order Zingiberales, is recognized as the largest family within the order and is predominantly distributed across tropical and subtropical regions (Sirirugsa 1999). Renowned for its medicinal, culinary, and aromatic uses, the family is widely represented throughout the Malesian bioregion, which includes Indonesia, Malaysia, Singapore, the Philippines, Brunei, and Papua New Guinea. With over 57 genera and more than 1600 species documented globally, the family reflects remarkable taxonomic diversity (Saensouk and Saensouk 2021). Among these, *Amomum* stands out for its value as a spice and its extensive use in traditional medicine (Xia et al. 2004; Lamxay Lamxay and Newman 2012). This genus is primarily found in India, Indochina, and Sumatra, with its distribution extending from the Himalayas to the southern and southeastern regions of Asia and with a few species reported in northern Australia and Papua New Guinea (Ye et al. 2018). In Peninsular Malaysia, a notable diversity of 150–180 ginger species from 23 genera has been recorded (Holttum 1950; Habsah et al. 2000; Mabberley 2008; Govaerts et al. 2015).

*Amomum* sensu lato (s.l.), established by Roxburgh (1820), belongs to the subfamily Alpinioideae and tribe Alpinieae. The use of *sensu lato* (s.l.) indicates a broader taxonomic sense that encompasses *Amomum* and its related genera, such as *Wurfbainia* and *Meistera*. In the economic context, *Amomum* also shares a close affinity with economically important spice crops within the Zingiberaceae, such as ginger (*Zingiber officinale*), turmeric (*Curcuma longa*), cardamom (*Elettaria cardamomum*), galangal (*Alpinia galanga* or ‘Lengkuas’; *A. officinarum*), and white ginger (*Kaempferia galanga* or ‘Cekur’) (de Boer et al. 2018). In Southeast Asia, *Amomum* species have long been used as a substitute for cardamom and are used as food condiments and in traditional remedies (Xia et al. 2004; Lamxay and Newman 2012; Leong-Škorničková and Newman 2015). They include epiphytic forms as well as small terrestrial species with few-bladed shoots that grow on the forest floor (Xia et al. 2004).

Despite its ecological and ethnobotanical importance, the taxonomy of *Amomum* s.l. has historically been problematic. Borneo hosts the greatest diversity, with 36 species documented (de Boer et al. 2018). Holttum’s (1950) morphological study in Peninsular Malaysia identified several key features, such as elongated pseudostems and inflorescences, the absence of involucre bracts, and a broad yellow-and-white labellum with small red markings, the latter being the most characteristic feature (Syazana et al. 2018). However, species identification remains challenging due to considerable morphological variation, particularly in inflorescence characteristics, which often bloom only once or twice a year (Negi et al. 2018; Nontasit et al. 2014). A taxonomic revision of the Zingiberaceae of Peninsular Malaysia and Singapore conducted by Holttum (1950) listed 19 species. Subsequently, Newman et al. (2004) recorded 22 *Amomum* species in their checklist for the flora of Malesia. Locally, *Amomum* species are generally tall, herbaceous plants found from lowlands to highlands, typically in the understories of tropical forests near streams or rivers, often growing in forest gaps, although some species also grow abundantly at forest margins, in disturbed habitats, or within secondary forests (Xia et al. 2004; Leong-Škorničková and Newman 2015).

Although morphological approaches have long been used, classification within *Amomum* s.l. remains challenging due to characteristic variation and morphological convergence, and a lack of comprehensive herbarium specimens containing both vegetative and reproductive material has hindered accurate species delimitation. These challenges, particularly the difficulty in collecting specimens during short flowering and fruiting periods, underscore the limitations of morphology-based taxonomy. As Holttum (1950) noted (cited by de Boer et al. 2018), when no single morphological character can reliably distinguish *Amomum* s.l. from closely related genera, molecular approaches offer a powerful alternative and become essential.

Phylogenetic relationships within the Zingiberaceae family have therefore been investigated using molecular markers such as internal transcribed spacer (ITS), *matK*, and *rbcL* (Xia et al. 2004; de Boer et al. 2018; Cui et al. 2019). Previous molecular phylogenetic studies of *Amomum* s.l. confirmed the genus to be paraphyletic (Kress et al. 2002; de Boer et al. 2018). A comprehensive re-evaluation of the genus and its allies by de Boer et al. (2018) led to a major taxonomic revision, resulting in significant changes to the classification of *Amomum* s.l. Three previously recognized genera – *Conamomum*, *Meistera*, and *Wurfbainia* – were resurrected, while three new genera, *Epiamomum*, *Lanxangia*, and *Sundamomum*, were described within this new generic framework.

This reclassification led to an updated list of *Amomum* species in Peninsular Malaysia, comprising 28 species, with one species excluded (*Sundamomum macroglossa*) and two species (*A. macrodons* and *A. cephalotes*) classified as ‘uncertain’ (de Boer et al. 2018). The revised classification now encompasses five genera: *Amomum* s.s. (14 species), *Conamomum* (six species), *Wurfbainia* (five species), and *Meistera* (three species). Additionally, approximately 30 species previously classified under the genus *Elettariopsis* (Saensouk and Saensouk 2021), originally described by Baker (1892), were incorporated into *Amomum* s.l. based on molecular evidence (Kress et al. 2007; de Boer et al. 2018; Saensouk and Saensouk 2021). Despite these advancements, resolution at the species level remains insufficient, especially among Peninsular Malaysian taxa, where morphological convergence is common. Thus, although considerable progress has been made, a comprehensive phylogenetic framework for *Amomum* s.l. is still lacking.

In this context, chloroplast phylogenomics is a particularly effective tool for resolving species-level relationships within *Amomum* s.l. The relatively conserved structure and slower mutation rates of chloroplast genes make them reliable for resolving evolutionary relationships across taxonomic levels (Gong et al. 2022). Additionally, the use of chloroplast genes in phylogenomic analyses has several advantages, including their uniparental inheritance, high copy number, and relatively conserved gene order (Zhou et al. 2021). Furthermore, chloroplast genes are involved in essential biological processes, such as photosynthesis and metabolism, making them particularly informative for understanding the evolution of plant lineages and their adaptation to diverse environments (Daniell et al. 2016). Overall, the inclusion of chloroplast genes in phylogenomic studies enhances the accuracy and resolution of evolutionary relationships among plant species, contributing to a better understanding of their evolutionary history and biodiversity.

Transcriptome-based analysis uses high-throughput RNA sequencing to reconstruct evolutionary relationships using expressed genes. Transcriptomic analysis allows the recovery of many expressed genes for multi-locus phylogenomic inference and offers insights into gene evolution, including chloroplast-related genes. Previous studies have demonstrated the utility of chloroplast markers in phylogenetic research, but plastome-level data for *Amomum* species of Peninsular Malaysia remain limited. A study by Gong et al. (2022) provides a comprehensive chloroplast genome analysis for *Amomum* s.l., offering valuable insights into their evolutionary history and species identification. However, this research does not include the Peninsular Malaysian taxa.

This gap in the existing literature underscores the need for broader taxon sampling, including Peninsular Malaysian species. The present study aims to fill this void by conducting a phylogenetic analysis of *Amomum* s.l. from Peninsular Malaysia based on transcriptome-derived chloroplast gene data. By analyzing chloroplast-related genes from the transcriptomes of selected *Amomum* s.l. species, this study aims to clarify their interspecific and intergeneric relationships, offering a clearer understanding of their evolutionary history. This research is expected to provide valuable insights into the taxonomy and classification of *Amomum* s.l., with broader implications for its conservation and ethnobotanical utilization.

## Methods

### Plant material

Fresh plant material of accessible and flowering *Amomum* s.l. specimens in Peninsular Malaysia was collected during targeted fieldwork. Field sampling was conducted at type localities identified from herbarium records or previously published sources, representing either the original collection sites or the nearest documented localities. Herbarium voucher specimens were prepared from portions of the collected material and deposited at the Kepong Herbarium (KEP), Forest Research Institute Malaysia.

A total of seven *Amomum* s.l. species were analyzed in this study, each represented by two biological replicates obtained from two clumps. The sampled species and their localities in Peninsular Malaysia were as follows: *Wurfbainia testacea* (Ridl.) Škorničk. & A.D.Poulsen (Batu Caves, Selangor), *Wurfbainia uliginosa* (J.Koenig) Giseke (Forest Research Institute Malaysia (FRIM), Selangor), *Amomum curtisii* (Baker) Škorničk. & Hlavatá (the foothills of Penang Hill, Penang), *Amomum smithiae* (Y.K.Kam) Škorničk. & Hlavatá, *Amomum trilobum* Gagnep., *Meistera aculeata* (Roxb.) Škorničk. & M.F.Newman, and *Amomum elan* (C.K.Lim) Škorničk. & Hlavatá (Penang Botanic Gardens, Penang) (Table 1).

**Table 1.** List of the seven *Amomum* s.l. species sampled from Peninsular Malaysia, including their collection localities, voucher accession numbers, and GPS coordinates.

Species	Location	Accession number	GPS coordinates
*Amomum testaceum* (syn. *W. testacea*)	Batu Caves, Selangor	FRI97708	3°14.00'N, 101°37.00'E, elevation: 210 ft
*Amomum uliginosum* (syn. *W. uliginosa*)	Forest Research Institute of Malaysia, Selangor	FRI69087	3°08.53'N, 101°24.78'E, elevation: 260 ft
*Amomum smithiae*	Penang Botanic Gardens, Penang	PBG 19005137	5°26.42'N, 100°17.32'E, elevation: 200 ft
*Amomum aculeatum* (syn. *M. aculeata*)	Penang Botanic Gardens, Penang	PBG 19005063	5°26.50'N, 100°17.12'E, elevation: 310 ft
*Amomum curtisii*	Penang Hill, Penang	PBG 19005181	5°25.15'N, 100°15.87'E, elevation: 300 ft
*Amomum trilobum*	Penang Botanic Gardens, Penang	PBG 20240120	5°26.50'N, 100°17.29'E, elevation: 280 ft
*Amomum elan*	Penang Botanic Gardens, Penang	PBG 19005014	5°26.50'N, 100°17.12'E, elevation: 200 ft

For each flowering specimen, species identity was confirmed prior to collection. Fresh leaves, stems, roots, and rhizomes were collected, rinsed in distilled water, dried, cut into small pieces, and immediately preserved in RNAlater solution (Thermo Fisher Scientific, USA). Samples were subsequently ground in liquid nitrogen and stored at –80 °C until further processing.

Tissues were harvested at equivalent developmental stages to ensure comparability across species. For example, the second leaf from the branch apex was consistently sampled, and stem tissues were collected from the same branch at an equivalent distance from the apex. Sampling was conducted between March and April 2022, during the flowering season, between 08:00 and 10:00 AM.

In this study, species belonging to the broadly circumscribed *Amomum* complex (*Amomum* s.l.), including taxa currently recognized under segregate genera such as *Wurfbainia* and *Meistera*, as mentioned above, are referred to using their original *Amomum* names for consistency with field records, sequence datasets, and analytical workflows (Table 1).

### RNA extraction and sequencing

RNA was extracted using the Macherey and Nagel NucleoSpin RNA Plant kit with minor modifications to the manufacturer’s protocol (Fareed and Singaram 2023). The RNA was subsequently used for cDNA library preparation and Illumina sequencing by Apical Scientific Sdn. Bhd., Malaysia. In addition, 93 accessions and two outgroups, *Hedychium coronarium*NC045863 and *Roscoea humeana*NC046582, were downloaded from the National Center for Biotechnology Information (NCBI) to provide a comprehensive phylogenomic analysis. The selected taxa are appropriate outgroups because they belong to distinct lineages within Zingiberaceae and fall outside the target group being studied.

### RNA sequencing, assembly, and annotation

RNA samples with a minimum RNA integrity number (RIN) value of 6.5, high purity ratios (A_260/280_ ~ 2.0), and acceptable A260/230 values ranging from 2.0 to 2.2 were selected to ensure high-quality RNA. These samples, derived from pooled leaf, stem, and root tissues for each of the seven *Amomum* species, were sent to Apical Scientific Sdn. Bhd., Malaysia, for cDNA library construction and Illumina RNA-seq. Prior to downstream analyses, raw reads were subjected to quality assessment using FASTQC (Bioinformatics 2011) to identify and eliminate low-quality reads. Cleaned reads were then assembled *de novo* using Trinity v2.6.6 (Grabherr et al. 2011).

### Partial chloroplast gene analysis

For visualization of gene expression across species, heatmaps were generated using FPKM values derived from the transcriptome assemblies. Expression matrices were normalized using log_2_ (FPKM + 1) transformation to stabilize variance and minimize the influence of highly expressed genes (Anders and Huber 2010; Conesa et al. 2016). Hierarchical clustering was performed using Euclidean distance and average linkage to reveal expression-based relationships among species, following approaches commonly applied in plant transcriptomics and plastid expression analyses (Wicke et al. 2011). Heatmaps were generated using Seaborn’s clustermap function in Python, where red indicates high expression and green indicates low expression. Genes with zero or missing values across all species were excluded from the final heatmap prior to the log_2_ (FPKM + 1) transformation.

Chloroplast gene maps were visualized using OrganellarGenomeDRAW (OGDRAW) v1.3.1 (Greiner et al. 2019). Gene coordinates were extracted from BLAST alignments of chloroplast reference genes against the transcriptome assemblies of each species. Nucleotide diversity (*π*) was calculated to assess the level of sequence polymorphism across the chloroplast regions of the seven *Amomum* species. A total of 43 genes were aligned using ClustalW in BioEdit v7.2.5 (Hall 1999) with default parameters. The resulting multiple sequence alignment served as input for sliding-window analysis in DnaSP v6.12.03 (Rozas et al. 2017), applying a window size of 600 bp and a step size of 200 bp to compute *π* values across the alignment. Nucleotide positions were plotted against *π* values to visualize regions of high and low diversity. Gene boundaries were annotated on the graph using custom Python scripts (Suppl. material 1) to facilitate the identification of polymorphism hotspots associated with specific chloroplast genes.

### Phylogenetic analyses

To complement the transcriptomic data, available complete chloroplast genomes of closely related species within the genera *Amomum* s.l., *Kaempferia*, *Alpinia*, and *Zingiber* were retrieved from NCBI (Suppl. material 1: table S4). Two additional species, namely *Hedychium coronarium*NC045863 and *Roscoea humeana*NC046582, were included as outgroups. Phylogenetic reconstruction was conducted using two commonly employed methods: Bayesian inference (BI) and maximum likelihood (ML) (Huelsenbeck and Ronquist 2001; Stamatakis et al. 2008).

Bayesian analysis was performed using MrBayes v3.2.6 on XSEDE (Huelsenbeck and Ronquist 2001) via the CIPRES portal on XSEDE. jModelTest2 was used to select the best-fit nucleotide substitution model for the dataset. The analysis used three substitution schemes, unequal base frequencies, gamma-distributed rate variation among sites with four rate categories, and Akaike information criterion (AIC) and AIC corrected for small samples (AICc) (de Boer et al. 2018). Both AIC and AICc selected GTR+G as the best-fit model. Each run consisted of three incrementally heated chains and one cold Markov chain Monte Carlo (MCMC) chain, with a temperature parameter of 0.2, executed for 1 × 10^6^ generations and sampled every 1000 generations. All other parameters were maintained at default settings. Resulting phylograms were visualized and edited in FigTree (Rambaut 2007) with outgroup rooting applied to produce a 50% majority-rule consensus tree showing Bayesian posterior probability values on branches. ML analysis was performed using RAxML version 8 on the CIPRES portal on XSEDE, using the GTRGAMMA model with 500 bootstrap replicates mapped onto the best-scoring tree (Stamatakis et al. 2008). The resulting ML tree was visualized using FigTree (Rambaut 2007).

## Results

### Partial chloroplast gene analysis

The transcriptomic sequencing results generated approximately 170 GB of raw data. A total of 85 chloroplast genes were identified in the seven *Amomum* s.l. species from Peninsular Malaysia. In this study, 43 genes were analyzed, and the remaining 42 genes were excluded because they were short sequences or incomplete. Table 2 shows a summary of sequence-variability information for the seven species, and Table 3 shows the genes found in all seven samples and their categories.

**Table 2.** Summary of sequence-variability information of seven *Amomum* s.l. species.

Type	*A. aculeatum*	*A. trilobum*	*A. uliginosum*	*A. elan*	*A. smithiae*	*A. curtisii*	*A. testaceum*
**Total length (bp)**	27414	27499	27499	27499	27499	27500	27500
**Number of genes**	43	43	43	43	43	43	43
**Protein coding genes**	43	43	43	43	43	43	43
**Total GC (%) content**	37.4	38	37.7	37.8	37.7	37.9	37.4
**CDS.AT1**	64.9	64.9	64.5	64.6	64.7	64.7	64.7
**CDS.AT2**	61	61	60.5	61.1	61	60.7	61
**CDS.AT3**	62	61.3	61	61.2	61.2	60.9	62

**Table 3.** Genes present in all seven species.

Gene category	Gene names
**Photosystem I**	*psaJ*
**Photosystem II**	*psbA*, *psbB*, *psbD*, *psbE*, *psbF*, *psbG*, *psbH*, *psbI*, *psbJ*, *psbK*, *psbM*, *psbN*, *psbZ*
**ATP synthase**	*atpA*, *atpF*, *atpH*, *atpI*
**NAD(P)H dehydrogenase**	*ndhE*, *ndhJ*, *ndhK*
**Cytochrome b6f**	*petA*, *petL*
**Ribosomal proteins**	*rpl2*, *rpl14*, *rpl16*, *rpl20*, *rpl33*, *rpl36*, *rps3*, *rps7*, *rps8*, *rps11*, *rps14*, *rps16*, *rps19*
**Other**	*cemA*, *clpP*, *ycf4*, *ycf9*, *ycf10*, *ycf68*, *rbcL*

**Figure 1.**
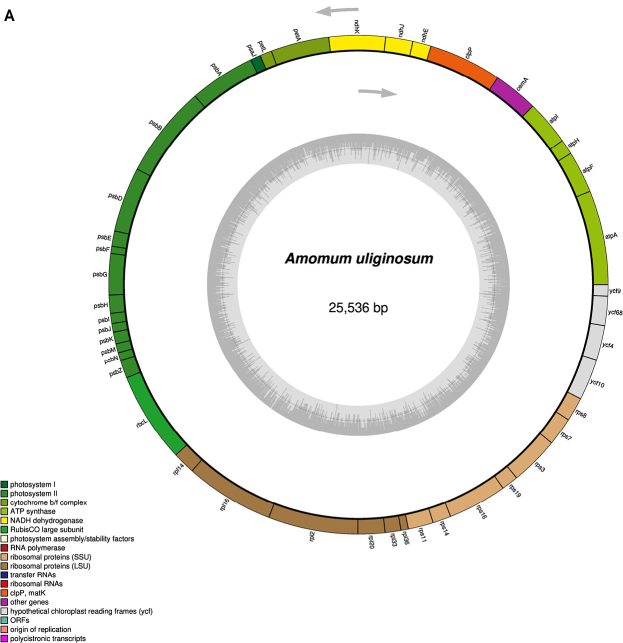
**A**. Partial chloroplast genes of *Amomum uliginosum*; **B**. Partial chloroplast genes of *Amomum trilobum*; **C**. Partial chloroplast genes of *Amomum testaceum*; **D**. Partial chloroplast genes of *Amomum smithiae*; **E**. Partial chloroplast genes of *Amomum elan*; **F**. Partial chloroplast genes of *Amomum curtisii*; **G**. Partial chloroplast genes of *Amomum aculeatum*.

The total length of the concatenated sequences ranged from 27,414 bp (*A. aculeatum*) to 27,500 bp (*A. curtisii* and *A. testaceum*). The overall GC content across species was relatively low, ranging from 37.4% to 38.0%. Across the seven *Amomum* species, A+T content was consistently elevated at all three codon positions of the chloroplast protein-coding genes. For all species, the first codon position exhibited the highest A+T content, ranging from 64.5% to 64.9%, while the second codon position showed the lowest, ranging from 60.5% to 61.1%, consistent with stronger evolutionary constraint at this position. The third codon position displayed moderately high A+T content, ranging from 60.9% to 62.0%, indicative of relaxed selective pressure and a higher likelihood of synonymous substitutions. Fig. 1A–G shows partial chloroplast genes of seven Peninsular Malaysian *Amomum* species.

**Figure 2.**
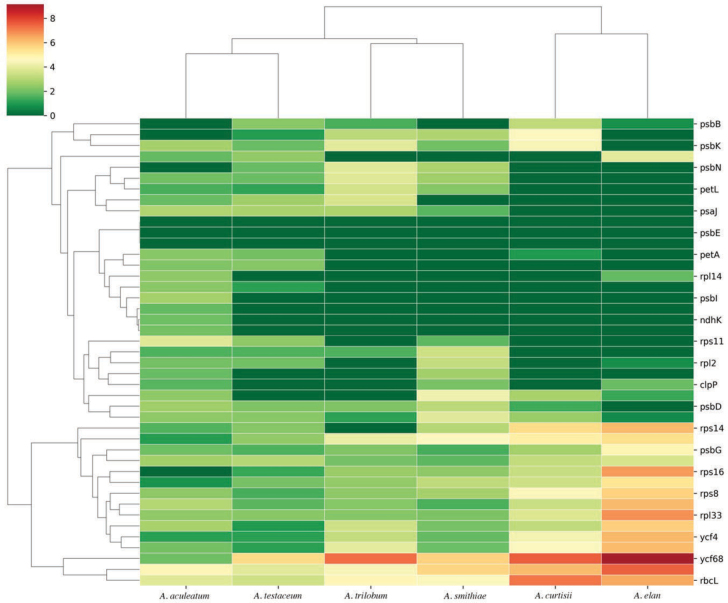
Hierarchical clustering heatmap of normalized (log_2_(FPKM + 1)) expression values of chloroplast genes across six *Amomum* species. The tree on the left indicates the hierarchical clustering dendrogram of the genes (rows), while the tree on top indicates the dendrogram of the species (columns).

The hierarchical clustering heatmap of normalized (log_2_(FPKM + 1)) chloroplast gene expression data for six *Amomum* species is shown in Fig. 2, and *A. uliginosum* was excluded due to poor alignment during gene expression quantification. Additionally, the elimination of genes with zero or nearly zero expression across all species was an essential filtering step in log-transformed transcriptome datasets to avoid artificial clustering biases. Both divergent and conserved gene expression patterns were identified across species. Several genes exhibited species-specific overexpression, but the majority of genes displayed moderate to low expression across species. Notably, genes *ycf68*, *ycf4*, *rpl33*, and *rps14* showed the highest expression levels in *A. elan*, whereas *A. aculeatum* and *A. trilobum* generally displayed lower expression levels across these genes. While *A. elan* formed a separate cluster, indicating a distinct chloroplast transcriptional landscape, *A. trilobum* and *A. testaceum* were closely clustered according to the clustering analysis, suggesting similar expression profiles. These trends highlight potential species-specific functional divergence within *Amomum* s.l. chloroplast genomes.

**Figure 3.**
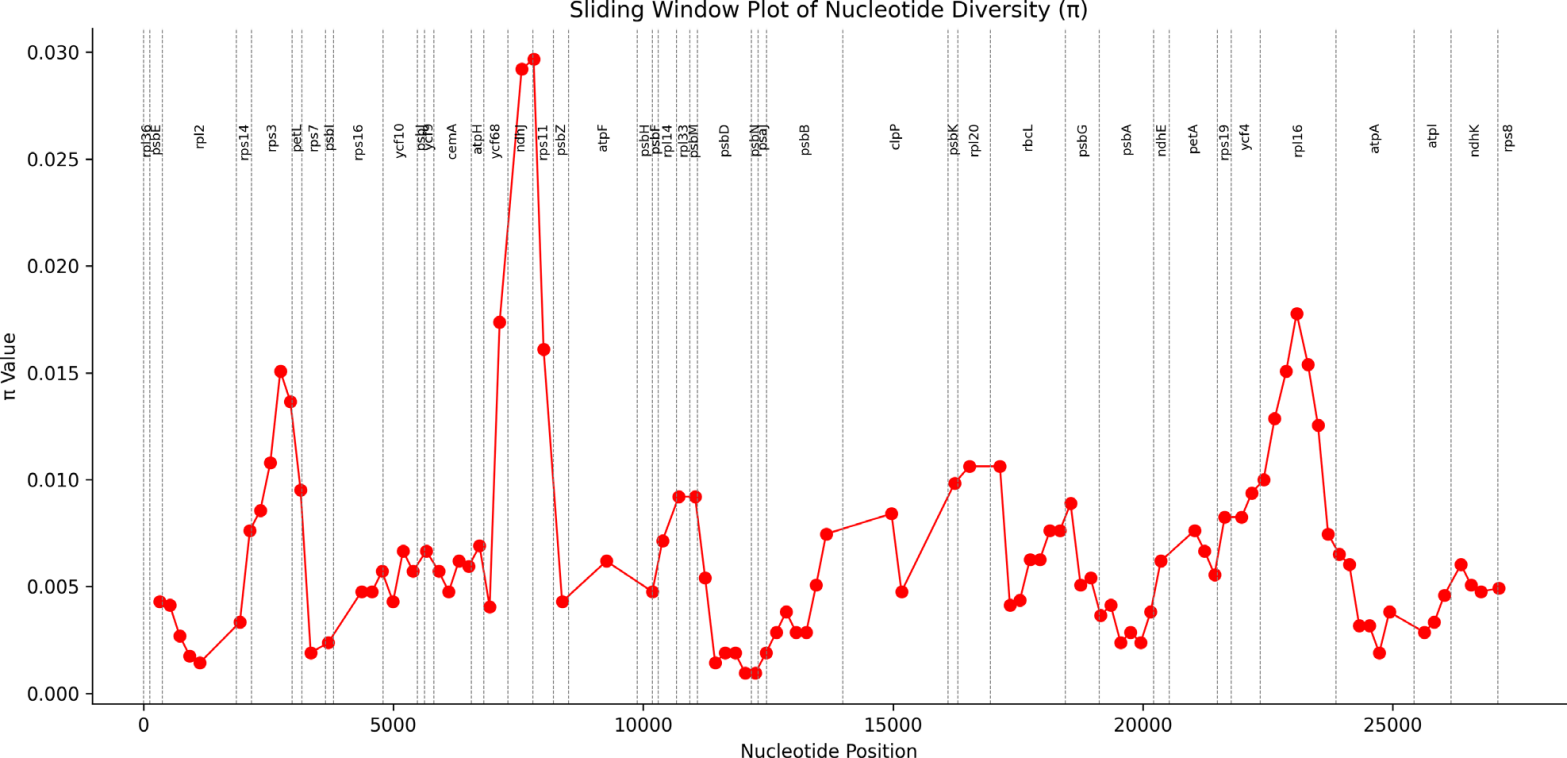
Nucleotide diversity and polymorphism hotspots from seven *Amomum* species showing regions of high and low sequence variation across the chloroplast genome.

Fig. 3 shows the results of the sliding-window analysis of nucleotide diversity (*π*) across the concatenated chloroplast genes. It demonstrated that the seven selected *Amomum* species have varying degrees of sequence polymorphism. Overall, the dataset showed moderate nucleotide variation, with *π* values ranging from 0 to approximately 0.030. The *ndhJ* gene region exhibited the highest nucleotide diversity, followed by elevated *π* values close to *psbT*, *psbZ*, and *rps14*. These areas might be hotspots for variation in the chloroplast and could be used as molecular markers for evolutionary research or species differentiation. Conversely, genes such as *rbcL*, *psbA*, and *atpI*, which are known to be highly conserved and functionally limited across plant lineages, were found in conserved regions with low *π* values. These patterns reflect a combination of evolutionary forces, whereby some genes accumulate mutations more frequently, such as *ndhJ*, *psbT*, and *rps14*, while others, such as *rbcL*, *psbA*, and *atpI*, remain highly conserved due to essential photosynthetic or housekeeping functions.

### Phylogenetic analysis

This study presents a comprehensive chloroplast phylogenomic analysis of the seven selected Peninsular Malaysian *Amomum* species, aimed at resolving their phylogenetic relationships within the family Zingiberaceae based on transcriptomic data. Although the *Amomum* s.l. chloroplast genome generally contains approximately 110–130 genes (Wu et al. 2017), only 43 chloroplast genes out of the 85 transcribed chloroplast genes were successfully extracted from the transcriptomic datasets and retained for subsequent analysis. BI and ML (RAxML) analyses were performed using 102 samples comprising 28,794 aligned positions and 43 concatenated chloroplast genes. Of these 102 samples analyzed, seven represented the Peninsular Malaysian species studied, 93 were related taxa downloaded from NCBI GenBank, and two were outgroups (*Hedychium coronarium*NC045863 and *Roscoea humeana*NC046582). Taxonomic names for reference sequences obtained from NCBI GenBank were retained according to their original database annotations and currently accepted generic classifications.

**Figure 4.**
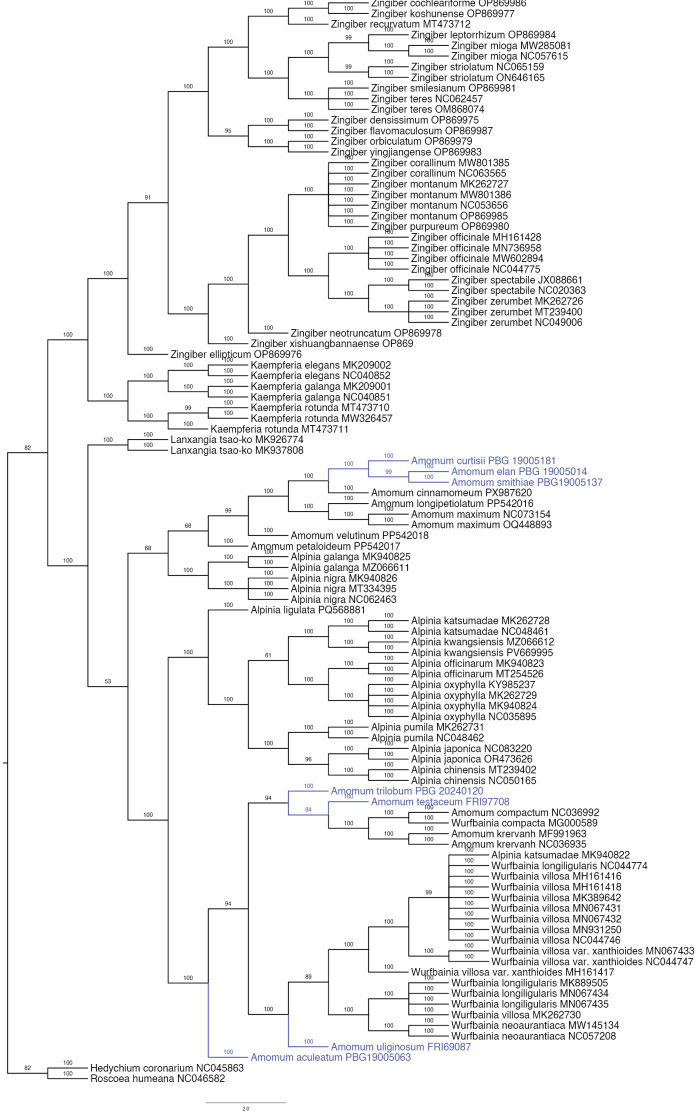
Bayesian inference phylogram showing evolutionary relationships among the sampled *Amomum* species.

**Figure 5.**
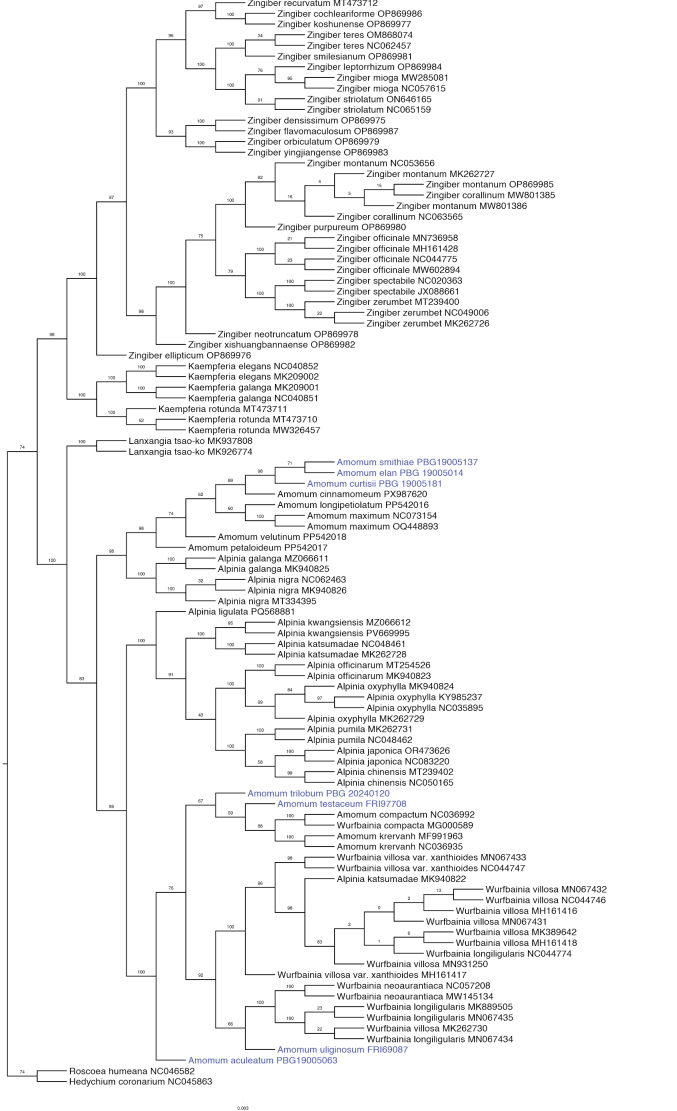
Maximum likelihood phylogram showing the evolutionary relationships among the sampled *Amomum* species.

The phylogenetic trees derived from both BI (Fig. 4) and ML analyses (Fig. 5) offer insights into the divergence and relatedness of these species and related genera. Both analyses revealed two major, well-supported clades, Clade A and Clade B, each with strong Bayesian posterior probability percentage (BPP) values of 100. Clade A primarily comprised species from the genera *Zingiber* and *Kaempferia* and was further divided into two well-supported clades. Subclade A1 (BPP = 100) included 21 *Zingiber* species, whereas subclade A2 (BPP = 100) consisted of three *Kaempferia* species. The *Kaempferia* subclade formed a sister lineage to the *Zingiber* species.

Clade B comprised taxa belonging to the *Amomum*–*Alpinia* complex, including representatives of *Amomum*, *Wurfbainia*, *Lanxangia*, and *Alpinia*. *Lanxangia tsao-ko* formed a strongly supported sister lineage to the remaining taxa within Clade B (BPP = 100). Subclade B1 showed moderate support (BPP = 68) and included three Peninsular Malaysian species, *A. curtisii*, *A. elan*, and *A. smithiae*, together with *Amomum longipetiolatum*, *A. cinnamomeum*, and *A. maximum*. Within this subclade, *A. curtisii* and *A. elan* clustered together with strong support (BPP = 100), while *A. smithiae* formed a sister lineage to this grouping. *Amomum velutinum* and *A. petaloideum* were resolved as sister species to subclade B1. These taxa collectively formed a sister clade to two *Alpinia* species: *Alpinia nigra* and *Alpinia galanga*.

Subclade B2 (BPP = 100) comprised the remaining *Alpinia* species and taxa associated with *Amomum* and *Wurfbainia*. *Alpinia ligulata* formed a sister lineage to two major clusters: one consisting of *A. katsumadae*, *A. kwangsiensis*, *A. officinarum*, and *A. oxyphylla* and the other comprising *A. pumila*, *A. japonica*, and *A. chinensis*. The *Alpinia* cluster formed a sister clade to taxa comprising *Amomum* and *Wurfbainia* species. Within this lineage, the two Peninsular Malaysian species *Amomum testaceum* (syn. *Wurfbainia testacea*) and *A. trilobum* formed a well-supported clade sister to *Amomum krervanh* (syn. *Wurfbainia vera* (Govaerts et al. 2021)) and *Amomum compactum* (syn. *Wurfbainia compacta*). In addition, *A. uliginosum* (syn. *Wurfbainia uliginosa*) and *A. aculeatum* (syn. *Meistera aculeata*) from Peninsular Malaysia formed a separate, highly supported clade (BPP = 100) positioned closely to *Wurfbainia villosa*, *W. longiligularis*, and *W. neoaurantiaca*.

The ML analysis using RAxML recovered a topology largely congruent with the Bayesian tree. Two major clades, A and B, were supported by strong bootstrap values of 98 and 100, respectively. Clade A was further divided into subclades A1 and A2, which were well resolved with bootstrap values of 100. The relationships among taxa within Clades A and B were generally consistent with those recovered in the Bayesian analysis, including the placement of the Peninsular Malaysian *Amomum* species. In both the BI and ML trees, all sampled Peninsular Malaysian *Amomum* species (highlighted in blue), except *A. trilobum* and *A. aculeatum* (syn. *Meistera aculeata*), were recovered within the *Amomum*–*Alpinia* lineage.

## Discussion

### Partial chloroplast gene analysis

The comparative chloroplast transcriptome analysis of *Amomum* species from Peninsular Malaysia revealed both conserved and divergent patterns in gene structure and expression. Gene exclusion is a common feature of transcriptome-derived plastid analyses and typically results from degraded RNA, incomplete assemblies, or low transcription abundance (Shi et al. 2012; Li et al. 2014). The overall structural conservation of the partial plastome was inferred based on the relatively constant length range of the concatenated genes (27,414–27,500 bp). The consistently low GC content observed in this study is characteristic of the A+T-biased plastid genomes of monocots (Morton 1998; Daniell et al. 2016). Codon position analyses further suggest purifying selection at the first codon position, stronger evolutionary constraint at the second, and relaxed selection at the third, enabling synonymous substitution and contributing to evolutionary plasticity (Zhang et al. 2006; Smith 2012).

Findings from the partial *Amomum* chloroplast genes analyzed here align with patterns reported in complete chloroplast genome studies. Jin et al. (2020) described the *A. villosum* plastome (~163.6–164.1 kb) as having a typical quadripartite structure with ~113 genes, supporting the structural stability and conserved gene content across *Amomum*. Similarly, Cui et al. (2019) emphasized both conserved inverted repeat (IR) boundaries and divergence in simple sequence repeat (SSR) content and non-coding regions across three medicinal *Amomum* species, highlighting features useful for species authentication. Although the present study does not capture the complete plastome, expression-based comparisons of conserved protein-coding genes correspond well with genome-level observations, emphasizing both overall structural conservation and gene-specific divergence.

The hierarchical heatmap derived from normalized chloroplast gene expression (log_2_(FPKM + 1)) illustrated distinct species-specific transcriptional landscapes across the six analyzed species, excluding *A. uliginosum*. Filtering out genes with zero or near-zero expression was essential to avoid artificial clustering biases; hence, not all 43 genes were included in the heatmap (Anders and Huber 2010). Despite the general pattern of moderate to low expression across species, several genes exhibited notable species-specific upregulation. Distinct clustering patterns suggest divergent plastid regulation, with *A. elan* showing a particularly unique expression profile. This may reflect lineage-specific retention or modulation of plastid ribosomal and *ycf* functions (Wakasugi et al. 1996; Ueda et al. 2007). The ribosomal gene *rpl33* is known to be critical for ribosome stability and translational initiation under cold stress (Tiller et al. 2012). Although less well understood, the *ycf14* and *ycf68* genes are frequently found in plastomes, with *ycf68* implicated in tRNA intron splicing in some lineages (Shinozaki et al. 1986; Raubeson and Jansen 2005).

Patterns of functional divergence are further supported by the clustering behavior observed. For instance, the close grouping of *A. trilobum* and *A. testaceum* (syn. *W. testacea*) suggests similarities in the regulation of plastid gene expression. In contrast, the distinct clustering of *A. elan* supports a trajectory of lineage-specific plastome expression evolution. Such variation in plastid gene expression may reflect differences in plastid gene loss and transfer, adaptation to ecological niches, or regulatory changes across lineages (Mohanta et al. 2020).

Nucleotide diversity (*π*) analysis revealed a wide range of polymorphisms across the chloroplast genomes of the Peninsular Malaysian *Amomum* species. The pronounced diversity peak at the *ndhJ* locus suggests relaxed selection or lineage-specific evolutionary pressure, positioning this region as a potential candidate for phylogeographic or DNA barcoding applications. Genes such as *ndhF*, *psbT*, and *rps14* have similarly been identified as informative markers for species discrimination and evolutionary inference in other plant families, including Zingiberaceae and Solanaceae (Shaw et al. 2007; Huang et al. 2014; Li et al. 2021). Conversely, genes such as *atpI*, *psbA*, and *rbcL* showed minimal variability, consistent with their highly conserved and essential functions in photosynthesis (Dong et al. 2012; Abdullah et al. 2020). The contrast between conserved and hypervariable regions highlights the dynamic evolutionary forces influencing the *Amomum* s.l. chloroplast genome and demonstrates the utility of sliding-window *π* analysis in identifying candidate molecular markers.

Previous work by Jin et al. (2020) and Cui et al. (2019) identified loci such as *ycf4*–*cemA* and other SSR-rich intergenic spacers as promising barcoding sites. The expression peaks observed in *rps14*, *rpl33*, *ycf14*, and *ycf68* in this study complement those findings. This further suggests that some variable protein-coding genes may also exhibit regulatory importance. Collectively, these findings highlight the value of integrating plastome structural features with transcriptome-based expression profiles to uncover candidate markers and gain insight into adaptive divergence within *Amomum* s.l.

Overall, this study demonstrates that chloroplast gene expression patterns in *Amomum* s.l. may be shaped by a combination of evolutionary constraint and species-specific regulatory shifts. Several genes, including *rps14*, *rpl33*, and *ycf68*, emerge as promising candidates for further functional studies aimed at understanding plastid function, adaptation, and diversification.

### Phylogenetic analysis

Phylogenomic analysis identified two major clades, with all Peninsular Malaysian *Amomum* species consistently placed within Clade B. This topology aligns with earlier classifications within the Zingiberaceae (Xia et al. 2004; Kress et al. 2002). The strong bootstrap support for the close relationship between *Zingiber* and *Kaempferia* further confirms previous taxonomic frameworks proposed by de Boer et al. (2018). In addition, the placement of *Lanxangia tsao-ko* as a sister lineage to the remaining taxa within Clade B further reinforces its recognition as a distinct lineage within the family, consistent with the findings of de Boer et al. (2018). Importantly, the present analyses further affirm the paraphyletic nature of *Amomum* s.l., as observed by de Boer et al. (2018) and Gong et al. (2022).

The present findings are broadly congruent with those of Gong et al. (2022), although several differences in species relationships were observed. Gong et al. (2022) identified *A. maximum* and *A. longipetiolatum* as sister taxa to *Alpinia nigra* and *A. galanga*, whereas the present study found *Amomum curtisii*, *A. elan*, and *A. smithiae* to be more closely related to *A. maximum* and *A. longipetiolatum*, together forming a sister lineage to the two *Alpinia* species. Hlavatá et al. (2023) previously grouped *A. trilobum* with *A. aff. elan*; however, the latter represents an uncertain taxonomic identification and may not accurately reflect the true placement of *A. elan*. In contrast, the present analysis clearly distinguishes *A. trilobum* and *A. elan* as distinct evolutionary lineages. These differences emphasize the influence of taxon sampling on phylogenetic resolution and highlight the importance of integrating both morphological and molecular datasets to refine species boundaries within the Zingiberaceae.

The phylogenetic relationships observed in this study also provide insight into the biogeographical history of the groups. Divergence patterns within Clade B, particularly the sister relationships between Peninsular Malaysian *Amomum* species and taxa distributed across other regions of Southeast Asia, suggest that historical geographical barriers may have influenced lineage diversification. This observation aligns with previous research indicating that sea-level fluctuations, mountain formation, and climatic changes have shaped dispersal and diversification patterns within the Zingiberaceae throughout Southeast Asia (Ashokan et al. 2022). The occurrence of closely related taxa across geographically distinct regions points to historical connectivity and subsequent regional diversification.

The taxonomic revisions proposed by de Boer et al. (2018) are also supported by the present findings. *Amomum uliginosum*, previously transferred to *Wurfbainia uliginosa* (J.Koenig) Škorničk. & A.D.Poulsen, clustered within the *Wurfbainia* lineage in the present phylogeny. Similarly, *Amomum testaceum* (syn. *Wurfbainia testacea*) resolved in close association with *A. compactum* (syn. *W. compacta*) and *A. krervanh* (syn. *W. vera*), further supporting recognition of *Wurfbainia* as a distinct lineage. Based on the present phylogeny, *A. trilobum* appears more closely associated with the *Wurfbainia* lineage than with the core *Amomum*–*Alpinia* cluster, suggesting that further taxonomic evaluation of this species is warranted. Similarly, *A. aculeatum* (syn. *Meistera aculeata*) resolved as a distinct lineage sister to the subclade consisting of *Amomum* and *Wurfbainia* species. However, this inference is currently limited by the availability of only a single representative sequence. The absence of complete plastome records for additional *Meistera* species in GenBank further restricts the phylogenetic resolution within this lineage and highlights the need for broader taxon sampling.

The consistent detection of paraphyly within *Amomum* s.l. across multiple phylogenetic studies (de Boer et al. 2018; Gong et al. 2022) raises important questions regarding the evolutionary history of this group. Such paraphyly may reflect complex processes such as incomplete lineage sorting, hybridization, or retention of ancestral polymorphisms, all of which warrant further investigation using expanded genomic datasets and broader taxonomic sampling.

Finally, it is important to acknowledge that phylogenies reconstructed based on individual or reduced chloroplast genes may differ from those inferred using complete plastome sequences. Whole-plastome analyses generally provide higher phylogenetic resolution owing to the inclusion of non-coding regions and additional informative sites. Nevertheless, analyses based on a reduced set of chloroplast genes, such as those used in the present study, were sufficient to recover robust phylogenetic patterns within the Peninsular Malaysian *Amomum* species.

## Conclusion

In summary, this study provides the first transcriptome-derived chloroplast phylogenomic framework for Peninsular Malaysian *Amomum* s.l. Using 43 conserved chloroplast protein-coding genes, the analyses consistently support the paraphyly of *Amomum* s.l. and align with recent taxonomic revisions within tribe Alpinieae. All sampled Peninsular Malaysian taxa were placed within Clade B, highlighting both well-supported and unresolved relationships that warrant further investigation using nuclear markers and complete plastome data. *Amomum trilobum* showed a closer association with *Wurfbainia* than with *Amomum*–*Alpinia*, suggesting the possible transfer of the species to *Wurfbainia* with further taxonomic evaluation.

Beyond phylogenetic structure, the study also reveals lineage-specific divergence in chloroplast gene expression. Variation in the expression of genes such as *rps14*, *rpl33*, and *ycf68* suggests potential functional shifts among species, complementing nucleotide diversity patterns that identify both conserved regions, e.g., *rbcL* and *psbA*, and hypervariable hotspots, e.g., *ndhJ*, *psbT*, and *rps14*. Together, these findings demonstrate the utility of transcriptome-derived chloroplast data for elucidating evolutionary histories where full plastomes are unavailable while also identifying promising candidate loci for future barcoding, phylogeographic, and functional studies.

## Additional information

### Artificial Intelligence (AI) use

The authors accept full responsibility for the content of the manuscript, including the disclosure of any use of AI.

Regarding the use of AI in the preparation of this manuscript, the authors declare the following: Microsoft Copilot.

Used for: Language, style and writing.

### Author ORCIDs

F.S. Fareed https://orcid.org/0000-0002-1090-6813

N. Singaram https://orcid.org/0000-0003-2608-7776

A.Z. Abdul Malek https://orcid.org/0009-0004-1724-3284

A.M. Hashim https://orcid.org/0000-0002-8320-0141

Y.Y. Sam https://orcid.org/0000-0001-8487-8029

O.I. Hin https://orcid.org/0009-0009-2855-6901

### Data availability

All of the data that support the findings of this study are available in the main text.

## Supplementary materials

Supplementary material 1 Accession numbers Data type xlsx https://binary.pensoft.net/file/1678446This dataset is made available under the Open Database License (http://opendatacommons.org/licenses/odbl/1.0/). The Open Database License (ODbL) is a license agreement intended to allow users to freely share, modify, and use this Dataset while maintaining this same freedom for others, provided that the original source and author(s) are credited. Fathmath Shaman Fareed, Nallammai Singaram, Ahmad Zuhairi Abdul Malek, Amalia Mohd Hashim, Yen Yen Sam, Ooi Im Hin

Supplementary material 2 Python scripts Data type py https://binary.pensoft.net/file/1678447This dataset is made available under the Open Database License (http://opendatacommons.org/licenses/odbl/1.0/). The Open Database License (ODbL) is a license agreement intended to allow users to freely share, modify, and use this Dataset while maintaining this same freedom for others, provided that the original source and author(s) are credited. Fathmath Shaman Fareed, Nallammai Singaram, Ahmad Zuhairi Abdul Malek, Amalia Mohd Hashim, Yen Yen Sam, Ooi Im Hin

## References

[B1] Abdullah, Mehmood F, Shahzadi I, Waseem S, Mirza B, Ahmed I, Waheed MT (2020) Chloroplast genome of *Hibiscus rosa-sinensis* (Malvaceae): Comparative analyses and identification of mutational hotspots. Genomics 112: 581–91. 10.1016/j.ygeno.2019.04.010 30998967

[B2] Anders S, Huber W (2010) Differential expression analysis for sequence count data. Genome Biology 11(10): R106. 10.1186/gb-2010-11-10-r106 PMC3218662 20979621

[B3] Ashokan A, Xavier A, Suksathan P, Ardiyani M, Leong-Škorničková J, Newman M, Kress WJ, Gowda V (2022) Himalayan orogeny and monsoon intensification explain species diversification in an endemic ginger (Hedychium: Zingiberaceae) from the Indo-Malayan Realm. Molecular Phylogenetics and Evolution 170: e107440. 10.1016/j.ympev.2022.107440 35192919

[B4] Baker JG (1892) *Amomum*. In: Hooker JD (Ed) The Flora of British India (Vol. 6). London, L Reeve Co., 233–243.

[B5] Bioinformatics B (2011) FastQC: A Quality Control Tool for High Throughput Sequence Data. Babraham Institute, Cambridge, UK.

[B6] Boer HD, Newman M, Poulsen AD, Droop AJ, Fér T, Thu Hiền LT, Hlavatá K, Lamxay V, Richardson JE, Steffen K, Leong-Škorničková J (2018) Convergent morphology in Alpinieae (Zingiberaceae): Recircumscribing *Amomum* as a monophyletic genus. Taxon 67(1): 6–36. 10.12705/671.2

[B7] Conesa A, Madriga P, Tarazona S, Gomez-Cabrero D, Cervera A, McPherson A, Szczesniak MW, Gaffney DJ, Elo LL, Zhang X, Mortazavi A (2016) A survey of best practices for RNA-seq data analysis. Genome Biology 17: 1–13. 10.1186/s13059-016-0881-8 PMC4728800 26813401

[B8] Cui Y, Nie L, Sun W, Xu Z, Wang Y, Yu J, Song J, Yao H (2019) Comparative and phylogenetic analyses of ginger (*Zingiber officinale*) in the family Zingiberaceae based on the complete chloroplast genome. Plants 8(8): e283. 10.3390/plants8080283 PMC6724139 31409043

[B9] Cui Y, Chen X, Nie L, Sun W, Hu H, Lin Y, Li H, Zheng X, Song J, Yao H (2019) Comparison and phylogenetic analysis of chloroplast genomes of three medicinal and edible *Amomum* species. International Journal of Molecular Sciences 20(16): e4040. 10.3390/ijms20164040 PMC6720276 31430862

[B10] Daniell H, Lin CS, Yu M, Chang WJ (2016) Chloroplast genomes: Diversity, evolution, and applications in genetic engineering. Genome Biology 17: e134. 10.1186/s13059-016-1004-2 PMC4918201 27339192

[B11] Dong W, Liu J, Yu J, Wang L, Zhou S (2012) Highly variable chloroplast markers for evaluating plant phylogeny at low taxonomic levels and for DNA barcoding. PLoS ONE 7(4): e35071. 10.1371/journal.pone.0035071 PMC3325284 22511980

[B12] Fareed FS, Singaram N (2023) A modified RNA extraction protocol for secondary metabolite rich *Amomum* species (Zingiberaceae). Malaysian Journal of Biochemistry and Molecular Biology 26(1): 51–58.

[B13] Gong L, Ding X, Guan W, Zhang D, Zhang J, Bai J, Xu W, Huang J, Qiu X, Zheng X, Zhang D (2022) Comparative chloroplast genome analyses of Amomum: Insights into evolutionary history and species identification. BMC Plant Biology 22(1): 1–17. 10.1186/s12870-022-03898-x PMC9644571 36352400

[B14] Govaerts R, Newman M, Lock JM (2015) World Checklist of Zingiberaceae. Royal Botanic Gardens, Kew. http://apps.kew.org/wcsp/ [Accessed 28 Jul 2025]

[B15] Govaerts R, Nic Lughadha E, Black N, Turner R, Paton A (2021) The world checklist of vascular plants, a continuously updated resource for exploring global plant diversity. Scientific Data 8(1): e215. 10.1038/s41597-021-00997-6 PMC8363670 34389730

[B16] Grabherr MG, Haas BJ, Yassour M, Levin JZ, Thompson DA, Amit I, Adiconis X, Fan L, Raychowdhury R, Zeng Q, Chen Z (2011) Trinity: Reconstructing a full-length transcriptome without a genome from RNA-Seq data. Nature Biotechnology 29(7): e644. 10.1038/nbt.1883 PMC3571712 21572440

[B17] Greiner S, Lehwark P, Bock R (2019) OrganellarGenomeDRAW (OGDRAW) version 1.3.1: Improved visualization of organelle genomes and annotation transfer. Nucleic Acids Research 47(W1): W59–W64. 10.1093/nar/gkz238 PMC6602502 30949694

[B18] Habsah M, Amran M, Mackeen MM, Lajis NH, Kikuzaki H, Nakatani N, Rahman AA, Ali AM (2000) Screening of Zingiberaceae extracts for antimicrobial and antioxidant activities. Journal of Ethnopharmacology 72(3): 403–410. 10.1016/S0378-8741(00)00223-3 10996279

[B19] Hall TA (1999) BioEdit: A user-friendly biological sequence alignment editor and analysis program for Windows 95/98/NT. Nucleic Acids Symposium Series 41: 95–98.

[B20] Huelsenbeck JP, Ronquist F (2001) MRBAYES: Bayesian inference of phylogenetic trees. Bioinformatics 17(8): 754–755. 10.1093/bioinformatics/17.8.754 11524383

[B21] Hlavatá K, Leong-Škorničková J, Záveská E, Šída O, Newman M, Mandáková T, Lysak MA, Marhold K, Fér T (2023) Phylogenomics and genome size evolution in *Amomum* s.s. (Zingiberaceae): Comparison of traditional and modern sequencing methods. Molecular Phylogenetics and Evolution 178: e107666. 10.1016/j.ympev.2022.107666 36384185

[B22] Holttum RE (1950) The Zingiberaceae of the Malay Peninsula. The Gardens’ Bulletin, Singapore 13(1): 1–249. 10.5281/zenodo.16664379

[B23] Huang H, Shi C, Liu Y, Mao SY, Gao LZ (2014) Thirteen *Camellia* chloroplast genome sequences determined by high-throughput sequencing: Genome structure and phylogenetic relationships. BMC Evolutionary Biology 14: e151. 10.1186/1471-2148-14-151 PMC4105164 25001059

[B24] Jin C, Li Z, Li Y, Wang S, Li L, Liu M, Ye J (2020) Transcriptome analysis of terpenoid biosynthetic genes and simple sequence repeat marker screening in *Eucommia ulmoides*. Molecular Biology Reports 47(3): 1979–1990. 10.1007/s11033-020-05294-w 32040708

[B25] Kress WJ, Prince LM, Williams KJ (2002) The phylogeny and a new classification of the gingers (Zingiberaceae): Evidence from molecular data. American Journal of Botany 89(10): 1682–1696. 10.3732/ajb.89.10.1682 21665595

[B26] Kress WJ, Newman MF, Poulsen AD, Specht C (2007) An analysis of generic circumscriptions in tribe Alpinieae (Alpinioideae: Zingiberaceae). Gardens’ Bulletin Singapore 59 (1&2): 113–128. 10.5281/zenodo.16485501

[B27] Lamxay V, Newman MF (2012) A revision of *Amomum* (Zingiberaceae) in Cambodia, Laos and Vietnam. Edinburgh Journal of Botany 69(1): 99–206. 10.1017/S0960428611000436

[B28] Leong-Škorničková J, Newman M (2015) Gingers of Cambodia, Laos & Vietnam. Singapore: Singapore Botanic Gardens, National Parks Board.

[B29] Li B, Fillmore N, Bai Y, Collins M, Thomson JA, Stewart R, Dewey CN (2014) Evaluation of de novo transcriptome assemblies from RNA-Seq data. Genome Biology 15(12): e553. 10.1186/s13059-014-0553-5 PMC4298084 25608678

[B30] Li X, Yang Y, Henry RJ, Rossetto M, Wang Y, Chen S (2021) Plant DNA barcoding: From gene to genome. Biological Reviews 96(2): 646–668. 10.1111/brv.12104 24666563

[B31] Mabberley DJ (2008) Mabberley’s Plant-Book: A Portable Dictionary of Plants, their Classifications and Uses (No. Ed. 3). Cambridge University Press. 10.1017/9781316335581

[B32] Morton BR (1998) Selection on the codon bias of chloroplast and cyanelle genes in different plant and algal lineages. Journal of Molecular Evolution 46(4): 449–459. 10.1007/pl00006325 9541540

[B33] Mohanta TK, Mishra AK, Khan A, Hashem A, Abd_Allah EF, Al-Harrasi A (2020) Gene loss and evolution of the plastome. Genes 11(10): e1133. 10.3390/genes11101133 PMC7650654 32992972

[B34] Negi BK, Joshi RK, Pandey A (2018) Status of large cardamom (*Amomum subulatum* roxb.) Farming systems in the changing scenario of modern economics of Sikkim, Himalaya. Global Journal of Bioscience and Biotechnology 7: 189–199.

[B35] Newman M, Lhuillier A, Poulsen AD (2004) Checklist of the Zingiberaceae of Malesia. Blumea. Supplement 16: 1–166.

[B36] Nontasit N, Kanlayanapaphon C, Mekanawakul M, Nualmangsar O (2014) Taxonomic studies and traditional uses of Zingiberaceae in Khao Luang National Park, Nakhon Si Thammarat Province, Thailand. Journal of Science and Technology (WJST) 12(8): 643–658. 10.14456/WJST.2015.64

[B37] Rambaut A (2007) FigTree, a graphical viewer of phylogenetic trees. (Computer Program). http://tree.bio.ed.ac.uk/software [accessed 28 July 2025]

[B38] Raubeson LA, Jansen RK (2005) Chloroplast genomes of plants. Plant Diversity and Evolution: 45–68. 10.1079/9780851999043.0045

[B39] Roxburgh W (1820) Plants of the Coromandel. Bulmer & Co, London, 300 pp.

[B40] Rozas J, Ferrer-Mata A, Sánchez-DelBarrio JC, Guirao-Rico S, Librado P, Ramos-Onsins SE, Sánchez-Gracia A (2017) DnaSP 6: DNA sequence polymorphism analysis of large datasets. Molecular Biology and Evolution 34(12): 3299–3302. 10.1093/molbev/msx248 29029172

[B41] Saensouk S, Saensouk P (2021) Diversity and cytological studies of the Genus *Amomum* Roxb. former *Elettariopsis Baker* (Zingiberaceae) in Thailand. Biodiversitas Journal of Biological Diversity 22(6). 10.13057/biodiv/d220624

[B42] Shaw J, Lickey EB, Schilling EE, Small RL (2007) Comparison of whole chloroplast genome sequences to choose noncoding regions for phylogenetic studies in angiosperms: The tortoise and the hare III. American Journal of Botany 94(2): 275–288. 10.3732/ajb.94.3.275 21636401

[B43] Shi C, Hu N, Huang H, Gao J, Zhao YJ, Gao LZ (2012) An improved chloroplast DNA extraction procedure for whole plastid genome sequencing. PLoS ONE 11(2): e0152164. 10.1371/journal.pone.0031468 PMC3285163 22384027

[B44] Shinozaki K, Ohme M, Tanaka M, Wakasugi T, Hayashida N, Matsubayashi T, Zaita N, Chunwongse J, Obokata J, Yamaguchi-Shinozaki K, Ohto C, Torazawa K, Meng BY, Sugita M, Deno H, Kamogashira T, Yamada K, Kusuda J, Takaiwa F, Kato A, Tohdoh N, Shimada H, Sugiura M (1986) The complete nucleotide sequence of the tobacco chloroplast genome: Its gene organization and expression. EMBO Journal 5(9): 2043–2049. 10.1002/j.1460-2075.1986.tb04464.x PMC1167080 16453699

[B45] Sirirugsa P (1999) Thai Zingiberaceae: Species diversity and their uses. Pure Applied Chemistry 70: 1–8.

[B46] Smith DR (2012) Updating our view of organelle genome nucleotide landscape. Frontiers in Genetics 3: e175. 10.3389/fgene.2012.00175 PMC3438683 22973299

[B47] Stamatakis A, Hoover P, Rougemont J (2008) A rapid bootstrap algorithm for the RAxML web servers. Systematic Biology 57: 758–771. 10.1080/10635150802429642 18853362

[B48] Syazana S, Meekiong K, Afifah N, Syauqina MY (2018) *Amomum bungoensis*: A new species of *Amomum* (Zingiberaceae) from Sarawak, Malaysia. Journal of Botany. 10.1155/2018/1978607

[B49] Tiller N, Weingartner M, Thiele W, Maximova E, Schöttler MA, Bock R (2012) The plastid-specific ribosomal proteins of *Arabidopsis thaliana* can be divided into non-essential and essential components of the ribosome. The Plant Journal 69(2): 302–316. 10.1111/j.1365-313X.2011.04791.x 21923745

[B50] Ueda M, Fujimoto M, Arimura SI, Murata J, Tsutsumi N, Kadowaki KI (2007) Loss of the rpl32 gene from the chloroplast genome and subsequent acquisition of a preexisting transit peptide within the nuclear gene in Populus. Gene 402(1–2): 51–56. 10.1016/j.gene.2007.07.019 17728076

[B51] Wakasugi T, Tsudzuki J, Sugiura M (1996) Loss of all ndh genes as determined by sequencing the entire chloroplast genome of the black pine Pinus thunbergii. Proceedings of the National Academy of Sciences of the United States of America 91(21): 9794–9798. 10.1073/pnas.91.21.9794 PMC44903 7937893

[B52] Wicke S, Schneeweiss GM, dePamphilis CW, Müller KF, Quandt D (2011) The evolution of the plastid chromosome in land plants: Gene content, gene order, gene function. Plant Molecular Biology 76(3–5): 273–297. 10.1007/s11103-011-9762-4 PMC3104136 21424877

[B53] Wu M, Li Q, Hu Z, Li X, Chen S (2017) The complete *Amomum kravanh* chloroplast genome sequence and phylogenetic analysis of the commelinids. Molecules 22(11): 1875. 10.3390/molecules22111875 PMC6150383 29104233

[B54] Xia YM, Kress WJ, Prince LM (2004) Phylogenetic analyses of Amomum (Alpinioideae: Zingiberaceae) using ITS and matK DNA sequence data. Systematic Botany 29(2): 334–344. 10.1600/036364404774195520

[B55] Ye XE, Leong‐Škorničková J, Xia NH (2018) Taxonomic studies on *Amomum* (Zingiberaceae) in China I: Introducing the subject and *Amomum velutinum* sp. nov. previously misidentified as *A. repoeense* and *A. subcapitatum*. Nordic Journal of Botany 36(5): njb-01661. 10.1111/njb.01661

[B56] Zhang Z, Li J, Zhao XQ, Wang J, Wong GKS, Yu J (2006) KaKs_Calculator: Calculating Ka and Ks through model selection and model averaging. Genomics, Proteomics & Bioinformatics 4(4): 259–263. 10.1016/S1672-0229(07)60007-2 PMC5054075 17531802

[B57] Zhou J, Zhang S, Wang J, Shen H, Ai B, Gao W, Zhang C, Fei Q, Yuan D, Wu Z, Tembrock LR (2021) Chloroplast genomes in *Populus* (Salicaceae): Comparisons from an intensively sampled genus reveal dynamic patterns of evolution. Scientific Reports 11(1): e9471. 10.1038/s41598-021-88160-4 PMC8096831 33947883

